# Self-selection in a population-based cohort study: impact on health service use and survival for bowel and lung cancer assessed using data linkage

**DOI:** 10.1186/s12874-018-0537-3

**Published:** 2018-08-08

**Authors:** Nicola Creighton, Stuart Purdie, Matthew Soeberg, Richard Walton, Deborah Baker, Jane Young

**Affiliations:** 1 Cancer Institute NSW, PO Box 41, Alexandria, Sydney, NSW 1435 Australia; 2 Asbestos Diseases Research Institute, Sydney, Australia; 3 Sax Institute, Sydney, Australia; 4 School of Public Health, University of Sydney and Sydney Local Health District, Sydney, Australia

**Keywords:** Cohort studies, Selection bias, Health care utilisation, Cancer, Sociodemographic factors

## Abstract

**Background:**

In contrast to aetiological associations, there is little empirical evidence for generalising health service use associations from cohort studies. We compared the health service use of cohort study participants diagnosed with bowel or lung cancer to the source population of people diagnosed with these cancers in New South Wales (NSW), Australia to assess the representativeness of health service use of the cohort study participants.

**Methods:**

Population-based cancer registry data for NSW residents aged ≥45 years at diagnosis of bowel or lung cancer were linked to the 45 and Up Study, a NSW population-based cohort study (N~ 267,000). We measured hospitalisation, emergency department (ED) attendance and all-cause survival, and risk factor associations with these outcomes using administrative data for cohort study participants and the source population. We assessed bias in prevalence and risk factor associations using ratios of relative frequency (RRF) and relative odds ratios (ROR), respectively.

**Results:**

People from major cities, non-English speaking countries and with comorbidites were under-represented among cohort study participants diagnosed with bowel (*n* = 1837) or lung (*n* = 969) cancer by 20–50%. Cohort study participants had similar hospitalisation and ED attendance compared with the source population. One-year survival after major surgical resection was similar, but cohort study participants had up to 25% higher post-diagnosis survival (lung cancer 3-year survival: RRF = 1.24, 95% confidence interval 1.12,1.37). Except for area-based socioeconomic position, risk factors associations with health service use measures and survival appeared relatively unbiased.

**Conclusions:**

Absolute measures of health service use and risk factor associations in a non-representative sample showed little evidence of bias. Non-comparability of risk factor measures of cohort study participants and non-participants, such as area-based socioeconomic position, may bias estimates of risk factor associations. Primary and outpatient care outcomes may be more vulnerable to bias.

**Electronic supplementary material:**

The online version of this article (10.1186/s12874-018-0537-3) contains supplementary material, which is available to authorized users.

## Background

Cohort studies have been established around the world to examine health and health care use in ageing populations [1–8]. Applying findings from these cohorts to health service policy and practice is vital to realising the public health benefit of these studies. Participants in cohort studies are typically healthier and more socioeconomically advantaged than the general population by self-selection or design (e.g. British Doctors Study). As a result, the prevalence of exposures and absolute risk of disease or death among cohort study participants are often different to their source population. While the basis for generalising aetiological associations from non-representative cohorts is well established [9], there is little empirical evidence for generalising health service use associations. Additionally, absolute measures of health service use are often required to inform health service policy and practice. The few studies examining the effect of self-selection on absolute measures of health service use are conflicting, finding higher [10, 11] and lower [12, 13] health service use among participants.

The 45 and Up Study is a population-based cohort study in New South Wales (NSW), Australia, that was established to improve knowledge of ageing, with health service use a priority area [14]. Cancer, a major ageing-associated disease, is the largest cause of burden of disease in Australia [15], is among leading causes in other high-income countries and is becoming a significant burden in middle- and low-income countries [16]. Providing effective, efficient and equitable access to cancer care services is important in reducing this burden. However, there is evidence that patterns of health service use, such as late diagnosis and reduced treatment uptake, across population subgroups lead to poorer cancer outcomes [17–19]. Demonstrating that patterns of health service use and associations with risk factors among cohort study participants are generalisable to the source population enables research findings from cohort studies to be applied with more confidence to health service policy and practice.

In this study, we aimed to assess differences in inpatient hospital use, emergency department attendance and survival among 45 and Up Study participants diagnosed with lung or bowel cancer compared with people diagnosed with these cancers aged 45 years and older in the NSW population using linked population-based cancer registry and administrative health data. We compared estimates of associations between risk factors (remoteness of residence, socioeconomic position, country of birth, comorbidity) and health service use and survival outcomes to assess selection bias.

## Methods

### Study design

This study used de-identified linked cancer registry, administrative hospital, death registry and 45 and Up Study cohort data. Around 267,000 NSW residents aged 45 years and older joined the Sax Institute’s 45 and Up Study between February 2006 and December 2009, representing around 10% of this age group. Participants were randomly selected from the Department of Human Services (formerly Medicare Australia) enrolment database, a national publicly funded universal health care scheme. People aged 80 years and older and those from rural areas were over-sampled by a factor of two and all remote residents were sampled. Participants were recruited by completing a postal questionnaire and consenting to follow-up and linkage of their health-related records. The response rate was reported as 18% mid-recruitment period [14] and additionally participants (< 1%) volunteered via a hotline.

Cancer case data were obtained from the NSW Cancer Registry (NSWCR), a statutory registry of all invasive cancer cases (excluding non-melanoma skin cancer) diagnosed in NSW residents. Admission records for all NSW public and private hospitals were obtained from the NSW Admitted Patient Data Collection. Emergency department (ED) attendances at public hospitals were obtained from the NSW Emergency Department Data Collection, which had substantively complete coverage of EDs in metropolitan areas but was incomplete for regional areas for the study period. Attendance data were not available for the small number (< 5) of EDs at private hospitals which made up < 5% of ED activity during the study period [20, 21]. Mortality follow-up was from deaths recorded on the NSW Registry of Birth Deaths and Marriages.

The study was conducted with ethical approval from the NSW Population and Health Services Research Ethics Committee (HREC/14/CIPHS/60). Probabilistic linkage of the datasets was conducted by the Centre for Health Record Linkage (CHeReL) with an estimated false positive rate of 5 per 1000 (www.cherel.org.au). Identifying information (such as names and addresses) was separated from content information in the datasets to protect privacy. The CHeReL uses Choicemaker software to match identifiers and create a de-identified Project Person Number that enables records for an individual to be ascertained across the study datasets by researchers without accessing identifying information. The 45 and Up Study is approved by the University of New South Wales Ethics Committee.

### Study population

People aged ≥45 years diagnosed with bowel cancer (International Classification of Diseases, 10th Edition, Australian Modification [ICD-10-AM] C18-C20) or non-small cell lung cancer (ICD-10-AM C34, excluding m8041-m8045 and m8246; hereafter ‘lung cancer’) between February 2006 (commencement of 45 and Up Study recruitment) and December 2012 (the most recent data available at the time of extraction) were ascertained from the NSWCR. Bowel and lung cancer were selected since they are commonly diagnosed cancers, are leading causes of cancer death and have high rates of health service use in Australia [22]. People with a cancer diagnosed prior to the index cancer (from January 2000 onwards) or with another cancer case diagnosed within three months of the index cancer were excluded. Cases of an uncommon histology type, notified to the NSWCR by death certificate only, or with an unknown diagnosis date or place of residence were excluded. Cancers with uncommon histology types were excluded since they have different treatment patterns and outcomes.

### Outcomes and study variables

We examined health service use in the year prior to and the year after diagnosis. We used measures of hospital use (number of overnight admissions and number of weeks in hospital, excluding hospitals that primarily provide sub- and non-acute care) and ED attendance since linked population data are available for these areas of health service use. We measured major resection (defined by the Australian Classification of Health Interventions) since surgery is the main curative treatment for bowel and lung cancer. We measured all-cause one- and three-year post-diagnosis survival and, for those who underwent resection, one-year post-operative survival. Survival outcomes were measured since there are high rates of health service use in the lead up to death [23]. Mortality follow-up was to September 2016.

Age at diagnosis, sex, area-based socioeconomic position (Index of Relative Socioeconomic Disadvantage [24] for Census Districts), remoteness of residence [25] and extent of disease at diagnosis were obtained from the NSWCR. Country of birth was obtained from the NSWCR for people diagnosed between 2006 and 2010 but was unavailable from the NSWCR for 2011–2012. Country of birth was obtained from hospital admission records for this period. Hospital type (public or private), urgency of admission and the Charlson comorbidity score [26] (calculated with a five year look-back from hospital-recorded diagnoses) were obtained from hospital admission records.

### Analysis

We compared demographic, cancer case and health service use characteristics by 45 and Up Study participation status using a ratio of relative frequency (RRF). This was calculated by dividing the proportions in the 45 and Up Study by the proportion in the NSW cancer population for each categorical variable [27, 28]. A ratio greater than one indicates over-representation and a ratio below one indicates under-representation among 45 and Up Study participants. We restricted the examination of risk factor associations to resection use, one-year post-diagnosis survival, > 4 weeks in hospital and > 2 ED attendances in the year after cancer diagnosis. We focused on examining potential bias in associations with remoteness of residence, socioeconomic position, country of birth and comorbidity since these factors are often the focus of health service use studies. Associations with these factors were examined using a multivariable logistic regression model including all the factors of interest and adjusting for factors with known prognostic importance. In the model of resection status, sex, age and extent of disease at diagnosis were included as prognostic factors. In the models of the other outcomes, resection status was included as an additional prognostic factor. Adjusted relative odds ratios (RORs) were calculated as the ratio of the OR of 45 and Up Study participants to the OR of the NSW cancer population. Confidence limits (CLs) for the RRFs and RORs were calculated using the formula described by Nohr et al. [28]. The formula assumes the subsample is a random sample of the population, which is not the case here; however, the coverage properties were found to be adequate in a similar study [28].

## Results

### Demographic and cancer characteristics

**Fig. 1:**
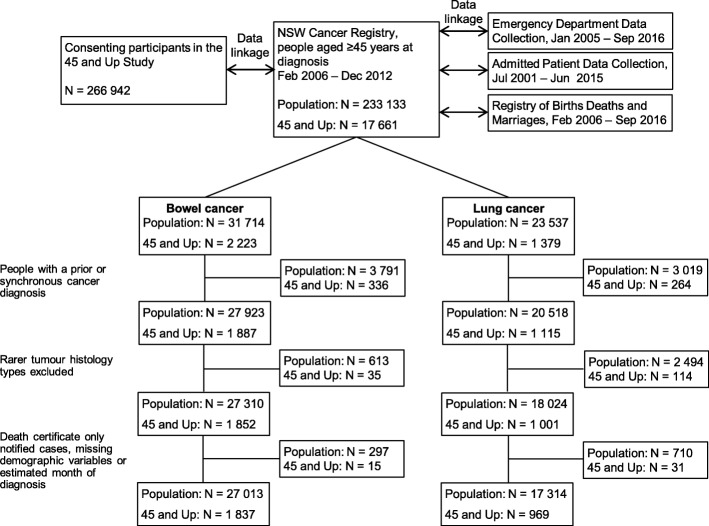
Study cohort

A total of 233,133 NSW residents aged ≥45 years were diagnosed with 245,266 cancer cases between February 2006 and December 2012 (Fig. 1). In NSW in 2008–12, the incidence per 100,000 age-standardised to the world population was 44.8 and 33.3 among men and 31.5 and 21.6 among women for bowel cancer and lung cancer respectively for all ages [29]. A total of 17,661 participants of the 45 and Up Study were diagnosed with cancer after enrolment, 7.6% of all NSW residents diagnosed. Lung cancer was under-represented among 45 and Up Study participants diagnosed with cancer (7.8% [*n* = 1379] v 10.1% [*n* = 23,537]; RRF = 0.77, CL 0.74, 0.81). In the final analysis cohorts, 6.8% (*n* = 1837) and 5.6% (*n* = 969) of NSW residents aged ≥45 years at diagnosis of bowel or lung cancer, respectively, were 45 and Up Study participants.

Sex and age distributions of 45 and Up Study participants diagnosed with bowel or lung cancer were similar to the NSW cancer population distributions (Table 1). Although there was under-representation of the youngest age groups, the median and interquartile ranges of age at diagnosis were similar. People from regional and remote areas were over-represented among 45 and Up Study participants by up to 50%. The distribution of socioeconomic position was similar between participants and the source population, particularly for bowel cancer. However, the over-representation of regional and remote areas among 45 and Up Study participants affects the distribution of socioeconomic position since these areas are generally more socioeconomically disadvantaged than major cities. Stratifying by remoteness, the over-representation of people from less disadvantaged areas was evident. For example in major cities, people diagnosed with bowel cancer from areas in the least disadvantaged socioeconomic quintile were over-represented in the 45 and Up Study (26.7% [*n* = 248] v 21.9% [*n* = 3918]; RRF 1.22, CL 1.10, 1.35) and the most disadvantaged quintile was under-represented (14.2% [*n* = 132] v 19.3% [*n* = 3452]; RRF 0.74, CL 0.63, 0.86) (see Additional file 1: Table 1). People from non-English speaking countries of birth were under-represented by a factor of two for both cancers. Extent of disease at diagnosis was similar, although the proportion of 45 and Up Study participants diagnosed with localised bowel cancer was slightly higher (34.7% [*n* = 637] v 31.4% [*n* = 8493]; RRF 1.10, CL 1.04, 1.17). 45 and Up Study participants had lower Charlson comorbidity scores for both cancers, with a higher proportion of 45 and Up Study participants with a score of zero and a lower proportion with a score of two or more.

**Table 1:** Cancer case characteristics of people diagnosed with bowel or lung cancer and ratio of relative frequency (RRF), 45 and Up Study participants and NSW residents aged ≥45 years

	Bowel cancer					Lung cancer				
	45 and Up		NSW			45 and Up		NSW		
	n	%	n	%	RRF (95% CL)	n	%	n	%	RRF (95% CL)
Sex										
Male	1009	54.9	14,585	54.0	1.02 (0.98,1.06)	545	56.2	10,177	58.8	0.96 (0.91,1.01)
Female	828	45.1	12,428	46.0	0.98 (0.93,1.03)	424	43.8	7137	41.2	1.06 (0.99,1.14)
Age at diagnosis										
45–54 years	117	6.4	2542	9.4	0.68 (0.57,0.80)	46	4.7	1403	8.1	0.59 (0.44,0.77)
55–64 years	352	19.2	5865	21.7	0.88 (0.81,0.97)	171	17.6	3578	20.7	0.85 (0.75,0.98)
65–74 years	601	32.7	8111	30.0	1.09 (1.02,1.16)	330	34.1	5579	32.2	1.06 (0.97,1.15)
75–84 years	549	29.9	7490	27.7	1.08 (1.01,1.15)	328	33.8	5157	29.8	1.14 (1.04,1.24)
85+ years	218	11.9	3005	11.1	1.07 (0.95,1.20)	94	9.7	1597	9.2	1.05 (0.87,1.27)
Median (IQR^a^)	72 (64–80)		71 (62–79)			73 (66–80)		71 (63–79)		
Charlson score										
0	1459	79.4	20,487	75.8	1.05 (1.02,1.07)	607	62.6	9662	55.8	1.12 (1.07,1.18)
1	138	7.5	2133	7.9	0.95 (0.81,1.11)	164	16.9	3131	18.1	0.94 (0.82,1.07)
2+	202	11.0	3585	13.3	0.83 (0.73,0.94)	136	14.0	3109	18.0	0.78 (0.67,0.91)
No admission	38	2.1	808	3.0	0.69 (0.51,0.94)	62	6.4	1412	8.2	0.78 (0.62,0.99)
Extent of disease										
Localised	637	34.7	8493	31.4	1.10 (1.04,1.17)	193	19.9	3190	18.4	1.08 (0.96,1.22)
Regional	748	40.7	11,495	42.6	0.96 (0.91,1.01)	223	23.0	3694	21.3	1.08 (0.96,1.21)
Distant	342	18.6	5314	19.7	0.95 (0.86,1.04)	440	45.4	7917	45.7	0.99 (0.93,1.06)
Unknown	110	6.0	1711	6.3	0.95 (0.79,1.13)	113	11.7	2513	14.5	0.80 (0.68,0.95)
Remoteness										
Major city	928	50.5	17,871	66.2	0.76 (0.73,0.80)	521	53.8	11,586	66.9	0.80 (0.76,0.85)
Inner regional	660	35.9	6735	24.9	1.44 (1.36,1.53)	336	34.7	4156	24.0	1.44 (1.33,1.57)
Outer regional & remote	249	13.6	2407	8.9	1.52 (1.36,1.70)	112	11.6	1572	9.1	1.27 (1.08,1.51)
Area-based socioeconomic position										
Least disadvantaged	290	15.8	4247	15.7	1.00 (0.91,1.11)	127	13.1	2078	12.0	1.09 (0.93,1.28)
Quintile 2	351	19.1	4777	17.7	1.08 (0.99,1.18)	151	15.6	2550	14.7	1.06 (0.92,1.22)
Quintile 3	397	21.6	5276	19.5	1.11 (1.02,1.20)	177	18.3	3268	18.9	0.97 (0.85,1.10)
Quintile 4	387	21.1	6178	22.9	0.92 (0.85,1.00)	255	26.3	4223	24.4	1.08 (0.97,1.19)
Most disadvantaged	412	22.4	6535	24.2	0.93 (0.85,1.01)	259	26.7	5195	30.0	0.89 (0.80,0.99)
Country of birth										
Australia	1370	74.6	18,110	67.0	1.11 (1.08,1.14)	682	70.4	11,027	63.7	1.11 (1.06,1.15)
Other English speaking	219	11.9	2542	9.4	1.27 (1.13,1.43)	135	13.9	2085	12.0	1.16 (0.99,1.35)
Non-English speaking	172	9.4	5411	20.0	0.47 (0.41,0.54)	122	12.6	3889	22.5	0.56 (0.48,0.66)
Unknown	76	4.1	950	3.5	1.18 (0.95,1.45)	30	3.1	313	1.8	1.71 (1.23,2.39)

^a^ IQR = interquartile range

*CL* confidence limits

### Hospital use, ED attendance and survival

Hospital use and ED attendance in the year prior to bowel or lung cancer diagnosis were similar for 45 and Up Study participants compared with the NSW bowel and lung cancer populations (Table 2). In the year after diagnosis, the number of hospital admissions and weeks in hospital were similar, although slightly fewer (RRF ~ 0.9) 45 and Up Study participants spent more than four weeks in hospital and a higher proportion of stays were in private hospitals. A higher proportion of 45 and Up Study participants had no ED attendances in the year after diagnosis, which was also the case for residents of major cities where coverage of ED attendances was complete (not shown). Emergency bowel resections were under-represented among 45 and Up Study participants (12.5% [*n* = 185] v 15.6% [*n* = 3324]; RRF 0.80, CL 0.70, 0.92). One year post-operative survival was similar for 45 and Up Study participants for both cancers (Table 3). One-year post-diagnosis survival was higher among 45 and Up Study participants by two and six percentage points for bowel and lung cancer respectively. Three-year post-diagnosis survival was around five percentage points higher among 45 and Up Study participants for both cancers, which for lung cancer is 24% higher than the population value (26.4% [*n* = 256] v 21.3% [*n* = 3686]; RRF 1.24, CL 1.12, 1.37).

**Table 2:** Hospital use and emergency department (ED) attendance in the year prior to and year following diagnosis of bowel or lung cancer and ratio of relative frequency (RRF), 45 and Up Study participants and NSW residents aged ≥45 years

	Bowel cancer					Lung cancer				
	45 and Up		NSW			45 and Up		NSW		
	n	%	n	%	RRF (95% CL)	n	%	n	%	RRF (95% CL)
Year prior to diagnosis										
Number of admission										
Zero	1419	77.2	20,480	75.8	1.02 (0.99,1.04)	695	71.7	12,332	71.2	1.01 (0.97,1.05)
1	270	14.7	4550	16.8	0.87 (0.78,0.97)	179	18.5	3275	18.9	0.98 (0.86,1.11)
2+	148	8.1	1983	7.3	1.10 (0.95,1.27)	95	9.8	1707	9.9	0.99 (0.83,1.20)
Number of weeks in hospital										
Zero	1418	77.2	20,444	75.7	1.02 (1.00,1.04)	694	71.6	12,300	71.0	1.01 (0.97,1.05)
1 week	276	15.0	4169	15.4	0.97 (0.88,1.08)	175	18.1	2936	17.0	1.07 (0.94,1.21)
> 1 week	143	7.8	2400	8.9	0.88 (0.75,1.02)	100	10.3	2078	12.0	0.86 (0.72,1.03)
Type of hospital										
0	191	28.8	1999	20.5	1.40 (1.23,1.60)	68	15.0	1155	14.4	1.04 (0.83,1.31)
1	472	71.2	7746	79.5	0.90 (0.82,0.97)	385	85.0	6862	85.6	0.99 (0.91,1.09)
Number of ED attendances										
Zero	1395	75.9	20,006	74.1	1.03 (1.00,1.05)	658	67.9	11,640	67.2	1.01 (0.97,1.05)
1	266	14.5	4523	16.7	0.86 (0.78,0.96)	183	18.9	3385	19.6	0.97 (0.85,1.10)
2+	176	9.6	2484	9.2	1.04 (0.91,1.19)	128	13.2	2289	13.2	1.00 (0.85,1.17)
Year following diagnosis										
Number of admissions										
Zero	159	8.7	2349	8.7	1.00 (0.86,1.15)	166	17.1	2849	16.5	1.04 (0.91,1.19)
1	825	44.9	11,532	42.7	1.05 (1.00,1.10)	370	38.2	6316	36.5	1.05 (0.97,1.13)
2	452	24.6	7171	26.5	0.93 (0.86,1.00)	220	22.7	4001	23.1	0.98 (0.88,1.10)
3+	401	21.8	5961	22.1	0.99 (0.91,1.08)	213	22.0	4148	24.0	0.92 (0.82,1.03)
Number of weeks in hospital										
Zero	141	7.7	1989	7.4	1.04 (0.89,1.21)	149	15.4	2452	14.2	1.09 (0.94,1.25)
Up to 2 weeks	975	53.1	13,172	48.8	1.09 (1.04,1.13)	409	42.2	6964	40.2	1.05 (0.98,1.13)
> 2 to 4 weeks	401	21.8	6489	24.0	0.91 (0.84,0.99)	231	23.8	4210	24.3	0.98 (0.88,1.09)
> 4 weeks	320	17.4	5363	19.9	0.88 (0.80,0.97)	180	18.6	3688	21.3	0.87 (0.77,0.99)
Type of hospital										
Private	1095	33.3	14,031	28.9	1.15 (1.10,1.21)	247	15.1	3833	12.5	1.20 (1.07,1.35)
Public	2192	66.7	34,527	71.1	0.94 (0.91,0.97)	1394	84.9	26,834	87.5	0.97 (0.94,1.00)
Number of ED attendances										
Zero	996	54.2	13,411	49.6	1.09 (1.05,1.14)	321	33.1	5195	30.0	1.10 (1.01,1.20)
1	395	21.5	6844	25.3	0.85 (0.78,0.92)	275	28.4	5138	29.7	0.96 (0.87,1.05)
2	195	10.6	3361	12.4	0.85 (0.75,0.97)	156	16.1	3118	18.0	0.89 (0.78,1.03)
3+	251	13.7	3397	12.6	1.09 (0.97,1.21)	217	22.4	3863	22.3	1.00 (0.90,1.12)
Resection										
No	362	19.7	5736	21.2	0.93 (0.85,1.01)	778	80.3	14,390	83.1	0.97 (0.94,1.00)
Yes	1475	80.3	21,277	78.8	1.02 (1.00,1.04)	191	19.7	2924	16.9	1.17 (1.03,1.32)
Urgency of resection^a^										
Planned/other	1290	87.5	17,953	84.4	1.04 (1.01,1.07)					
Emergency	185	12.5	3324	15.6	0.80 (0.70,0.92)					

^a^Results not shown for lung cancer since emergency resection is uncommon (< 2%) and there are fewer than five 45 and Up Study participants with an emergency resection

*CL* confidence limits

**Table 3:** Survival outcomes following diagnosis of bowel or lung cancer and ratio of relative frequency (RRF), 45 and Up Study participants and NSW residents aged ≥45 years

	Bowel cancer					Lung cancer				
	45 and Up		NSW			45 and Up		NSW		
	n	%	n	%	RRF (95% CL)	n	%	n	%	RRF (95% CL)
One-year post-operative survival										
No	140	9.5	2258	10.6	0.89 (0.77,1.04)	25	13.1	366	12.5	1.05 (0.72,1.52)
Yes	1335	90.5	19,019	89.4	1.01 (0.99,1.04)	166	86.9	2558	87.5	0.99 (0.87,1.14)
One-year post-diagnosis survival										
No	273	14.9	4646	17.2	0.86 (0.78,0.96)	506	52.2	10,082	58.2	0.90 (0.85,0.95)
Yes	1564	85.1	22,367	82.8	1.03 (1.01,1.05)	463	47.8	7232	41.8	1.14 (1.07,1.22)
Three-year post-diagnosis survival										
No	508	27.7	8716	32.3	0.86 (0.80,0.92)	713	73.6	13,628	78.7	0.93 (0.90,0.97)
Yes	1329	72.3	18,297	67.7	1.07 (1.04,1.10)	256	26.4	3686	21.3	1.24 (1.12,1.37)

*CL* confidence limits

### Associations between risk factors and outcomes

**Fig. 2:**
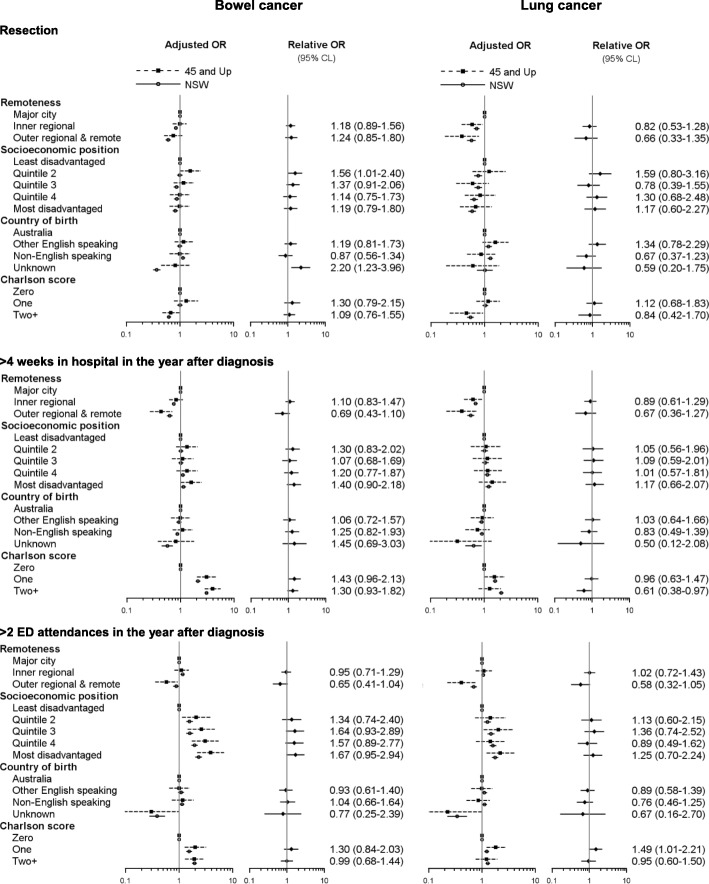
Adjusted* odds of resection, > 4 weeks in hospital and > 2 ED visits in the year after diagnosis for people diagnosed with bowel or lung cancer, 45 and Up Study participants and NSW residents aged *≥45* years. *Adjusted for sex, age at diagnosis, extent of disease at diagnosis, and additionally for hospital use and ED attendance outcomes, resection status

**Fig. 3:**
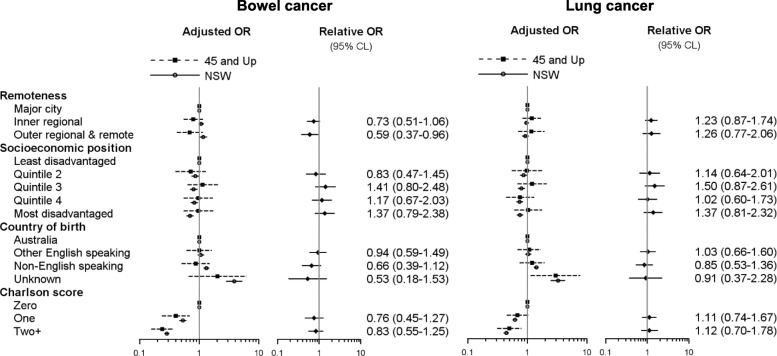
Adjusted* odds of one-year survival for people diagnosed with bowel or lung cancer, 45 and Up Study participants and NSW residents aged ≥ 45 years. *Adjusted for sex, age at diagnosis, extent of disease at diagnosis and resection status

There was little evidence of systemic bias in the estimates of associations between health service use and survival outcomes and remoteness of residence, country of birth and Charlson comorbidity score with odds ratios generally in the same direction and of similar magnitude among 45 and Up Study participants and the NSW population (Figs. 2 and 3). However, there are examples of odds ratios for 45 and Study participants and the NSW population being in the opposite direction for people born in non-English speaking countries (odds of > 4 weeks in hospital and surviving one year after diagnosis of bowel cancer) and for people from outer regional and remote areas (odds of surviving one year after diagnosis of bowel cancer). There are examples of the magnitude of odds ratios for 45 and Study participants and the NSW population differing (lower odds of > 4 weeks in hospital for Charlson score of 2+ and higher odds of > 2 ED attendances for Charlson score of 1 for 45 and Up Study participants diagnosed with lung cancer). Multivariable adjustment did not substantially change odds ratio estimates for risk factors (see Additional file 1: Tables).

In the NSW population, greater socioeconomic disadvantage was associated with lower odds of resection and one-year survival for bowel cancer whereas there was little evidence of an effect among 45 and Up Study participants from the point estimates of the disadvantage quintiles, although confidence intervals were wide. Odds of > 2 ED attendances in the year after bowel cancer diagnosis were in the same direction for 45 and Up Study participants as for the NSW population but were consistently around 1.5 times higher for the disadvantage quintiles. The relative consistency of differences in the magnitude and direction of odds ratio estimates for socioeconomic position across multiple outcomes among 45 and Up Study participants with bowel cancer could be indicative of bias.

## Discussion

The expectation of cohort study participants being healthier and wealthier than their source population was met in regard to health but was not as straightforward for wealth. The marginal distribution of socioeconomic position of 45 and Up Study participants diagnosed with bowel or lung cancer was similar to the source population of people aged ≥45 years diagnosed with these cancers. However, the expected over-representation of people from more socioeconomically advantaged areas was evident when stratified by remoteness. We attribute the difference between the stratified and marginal distributions of socioeconomic position to the over-representation of people from regional and remote areas. People from regional and remote areas were over-sampled in the design of 45 and Up Study to facilitate examining effects of rurality [14] and these areas are generally more socioeconomically disadvantaged than major cities [24].

Slightly more 45 and Up Study participants diagnosed with bowel or lung cancer had no comorbidity, with participants having higher post-diagnosis survival compared with the population. Lung cancers were less common among 45 and Up Study participants compared to the NSW population. Since most lung cancers are smoking-related [30], this likely reflects the lower prevalence of smokers and greater proportion of never smokers in the 45 and Up Study compared to NSW population survey-based estimates at baseline (7.4 and 12% smoking prevalence [31], 56% [32] and 40–50% never smokers [33] in the 45 and Up Study and NSW population respectively). A higher proportion of 45 and Up Study participants were diagnosed with localised bowel cancer compared to the NSW population, which may be related to 45 and Up Study participants having higher rates of bowel screening compared to NSW population estimates [31]. A national government-funded screening program was phased in from late 2006 to facilitate early detection of bowel cancer. Additionally screening tests have been available from pharmacies and medical practitioners. In contrast, lung cancer does not have a screening program and the diagnosis of localised lung cancer was similar between 45 and Up Study participants and the NSW population. Despite these differences, absolute measures of hospital and emergency department use in the year prior to and after cancer diagnosis were similar to the population estimates.

Estimates of risk factor associations among 45 and Up Study participants were generally consistent with population estimates, despite participants not being a representative sample in terms of these factors. While this is a demonstration of representativeness not being required for associations to be generalisable, the converse, that representativeness does not guarantee generalisability, was also demonstrated. The only risk factor that showed evidence of systemic bias was socioeconomic position among people with bowel cancer, which had a similar marginal distribution to the population. This apparent bias may in part be due to differing effects of socioeconomic disadvantage on health care utilisation in urban and rural settings. As in most epidemiological studies, the measure of socioeconomic position used in this study is a general index that may not capture contextual effects of disadvantage in urban and rural settings [24, 34]. The apparent bias may also be due to the area-level measure of socioeconomic position used in this study since an individual-level measure was not available in the population cancer data. 45 and Up Study participants may have different individual-level socioeconomic characteristics to those in the same area, making participants not comparable to non-participants. Similarly, since the 45 and Up Study baseline questionnaire was only available in English, country of birth associations were measured among people with sufficient English proficiency to respond which could have contributed to instances of non-English speaking country of birth associations being in the opposite direction to the population estimates.

Selection bias can occur when there are joint risk factors for study participation and outcomes and, furthermore, the magnitude of bias depends on the strength of these associations [35]. Health service use studies may be prone to selection bias since factors such as health literacy and health-seeking behaviours are likely to be associated with participation in a cohort study and are associated with health service use [36, 37]. Selection bias can be minimised by including factors associated with selection and outcome in adjustment models [35]. However, there are no questions on health literacy and few questions on health-seeking behaviours in the 45 and Up Study. It would be beneficial for cohort studies established with an aim of examining health service use to include validated measures of health literacy and health-seeking behaviours.

In aetiological studies, a key consideration in assessing the generalisability of associations is whether the underlying biological mechanisms are the same in participants and non-participants [38]. In health service use studies, non-biological mechanisms such as attitudes and beliefs towards health service use also need to be considered. In other studies, hospital use by responders to a health survey was similar to non-responders but out-of-hospital health service use differed. [10, 11] Hospital use is potentially less likely to be impacted by a person’s health-seeking propensity than out-of-hospital care since admitting physicians act as gatekeepers. Much activity for the early detection and diagnosis of cancer occurs in the primary care and outpatient settings. Health service use in response to cancer symptoms depends not only on clinical factors, but also psychosocial factors such as knowledge of symptoms and fear of cancer [39, 40]. Population-level primary care and outpatient data are not available for linkage studies in NSW. Health service use in these settings may be more vulnerable to the impacts of self-selection and requires further examination.

With the large number of comparisons in this study, some differences between estimates from 45 and Up Study participants and the NSW population are likely to occur by chance. Additionally, the 45 and Up Study participants were a small sample of the population and differences could result from sampling error rather than non-sampling error such as self-selection. The precision of the study estimates was limited by the small number of 45 and Up Study participants diagnosed with cancer. For individual cancer sites, even large cohort studies may be underpowered for the detection of differences between risk groups for health service use outcomes [41]. Furthermore, small numbers can reduce the number of confounders able to be included in adjustment models due to sparse-data bias [42]. The number of cancer cases diagnosed among 45 and Up Study participants will increase with longer follow-up. However, the findings of health service use studies using cancer cases diagnosed over long time periods may have limited applicability to health service policy and practice which often require timely data.

There are few studies examining the impact of self-selection on health service use outcomes and none focusing on cancer that we are aware of. Of these studies, most have examined participation in surveys with response rates of 50–80% conducted in Scandinavia or the Netherlands with one US Study [10, 11, 13, 43, 44]. The effect of self-selection on hospitalisation and psychiatric care has been examined in one cohort study [12] which had participation rates of 65–90% compared with the 45 and Up Study (18%) [14]. These studies have focussed on absolute measures of health service use and have reported both higher [10, 11, 44] and lower [12, 13, 43] health service use among participants. One study reported that health service use was only slightly (3–6%) lower among survey participants compared with all non-responders, but for the subset of people who did not respond due to illness there were much greater differences in health service use [43]. Similar to our study, the one study examining associations between demographic factors and health service use (including use of prescription drugs, hospitalisations, specialist, allied and dental care) among responders to a health survey found estimates were similar to those measured from target sample [10]. Our study complements another study on the representativeness of 45 and Up Study cohort which demonstrated the generalisability of aetiological associations measured from 45 and Up Study participants to survey-based NSW population estimates [31].

## Conclusions

This study contributes to the empirical evidence base for generalising health service use associations measured from non-representative samples. There was little evidence of bias in risk factor associations for the cancers and outcomes examined. However, the comparability of participants and non-participants with respect to the risk factor measure requires consideration. Further study is warranted on health service use in the primary and outpatient settings since the potential for selection bias is greater.

## Additional file

Additional file 1: Socioeconomic position by rurality and univariable and multivariable models of health service use outcomes. The additional file contains ratios of relative frequencies for area-based socioeconomic position stratified by rurality (major city; regional and remote) and univariable and multivariable logistic regression models of health service use outcomes (resection; > 4 weeks in hospital; > 2 emergency department attendances; one-year all-cause post-diagnosis survival) for 45 and Up Study participants and NSW residents aged ≥45 years at diagnosis of bowel or lung cancer. (DOCX 610 kb)

## Abbreviations

CL: Confidence limit
ED: Emergency department
ICD-10-AM: International Classification of Diseases, 10th Edition, Australian Modification
NSW: New South Wales
NSWCR: NSW Cancer Registry
ROR: Relative odds ratio
RRF: Ratio of relative frequency
