# Supplementary material for: Self-selection in a population-based cohort study: impact on health service use and survival for bowel and lung cancer assessed using data linkage
Source: BMC Med Res Methodol. 2018 Aug 8;18:84. doi: 10.1186/s12874-018-0537-3 (PMC6083588; doi:10.1186/s12874-018-0537-3)

**Table 1. Area-based socioeconomic position by rurality for people diagnosed with bowel or lung cancer and ratio of relative frequency (RRF, 95% confidence limits), 45 and Up Study participants and NSW residents aged ≥45 years**


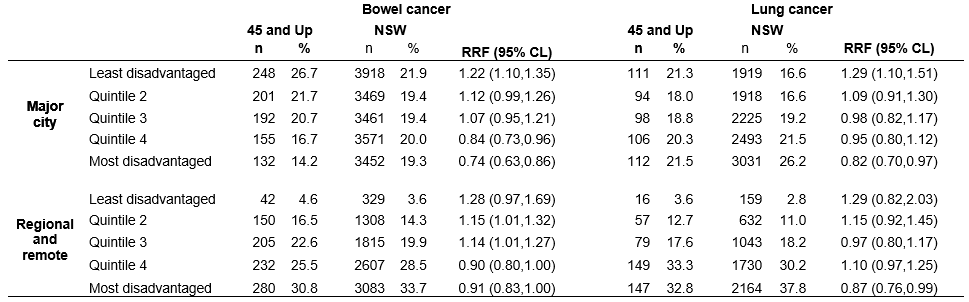


**Table 2: Univariable, multivariable adjusted and relative odds ratios (ROR, 95% confidence limits) of odds of resection after a diagnosis of bowel cancer, 45 and Up Study participants and NSW residents aged ≥45 years**


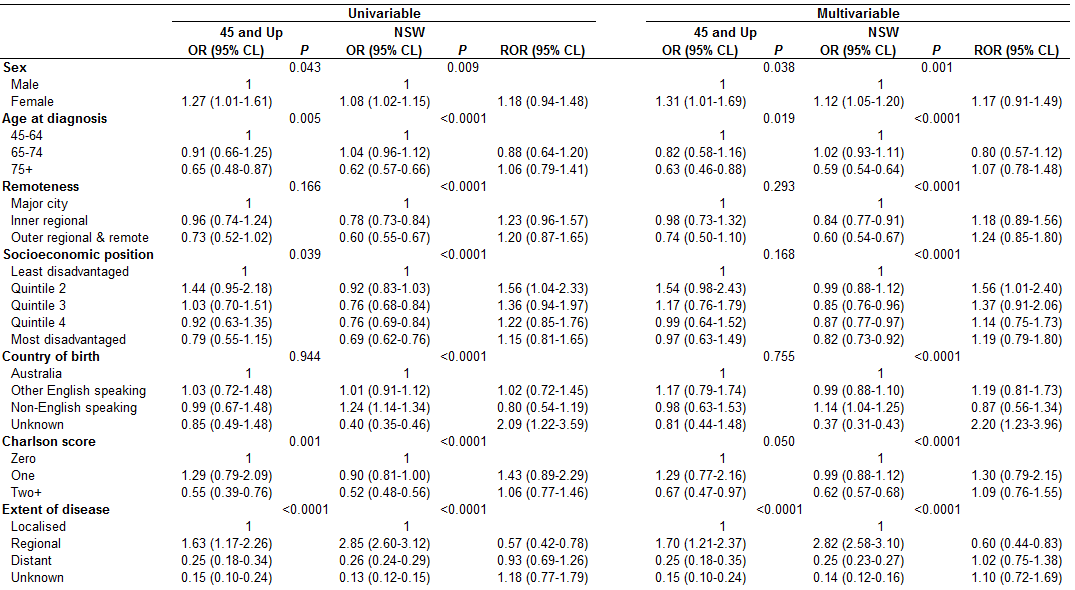


**Table 3: Univariable, multivariable adjusted and relative odds ratios (ROR, 95% confidence limits) of odds of resection after a diagnosis of lung cancer, 45 and Up Study participants and NSW residents aged ≥45 years**


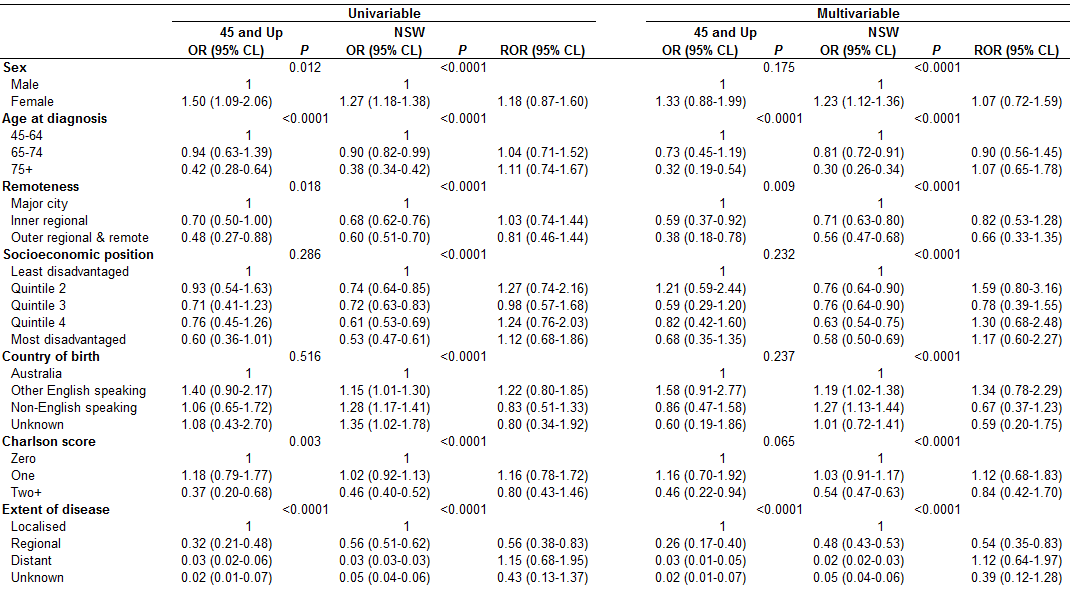


**Table 4: Univariable, multivariable adjusted and relative odds ratios (ROR, 95% confidence limits) of odds of >4 weeks in hospital in the year after a diagnosis of bowel cancer, 45 and Up Study participants and NSW residents aged ≥45 years**


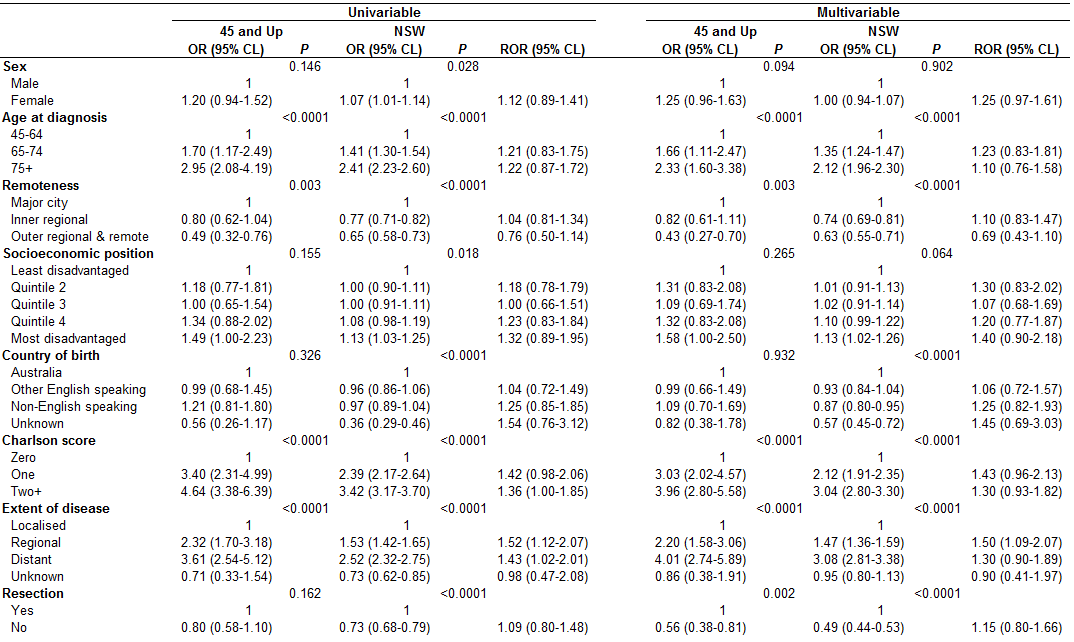


**Table 5: Univariable, multivariable adjusted and relative odds ratios (ROR, 95% confidence limits) of odds of >4 weeks in hospital in the year after a diagnosis of lung cancer, 45 and Up Study participants and NSW residents aged ≥45 years**


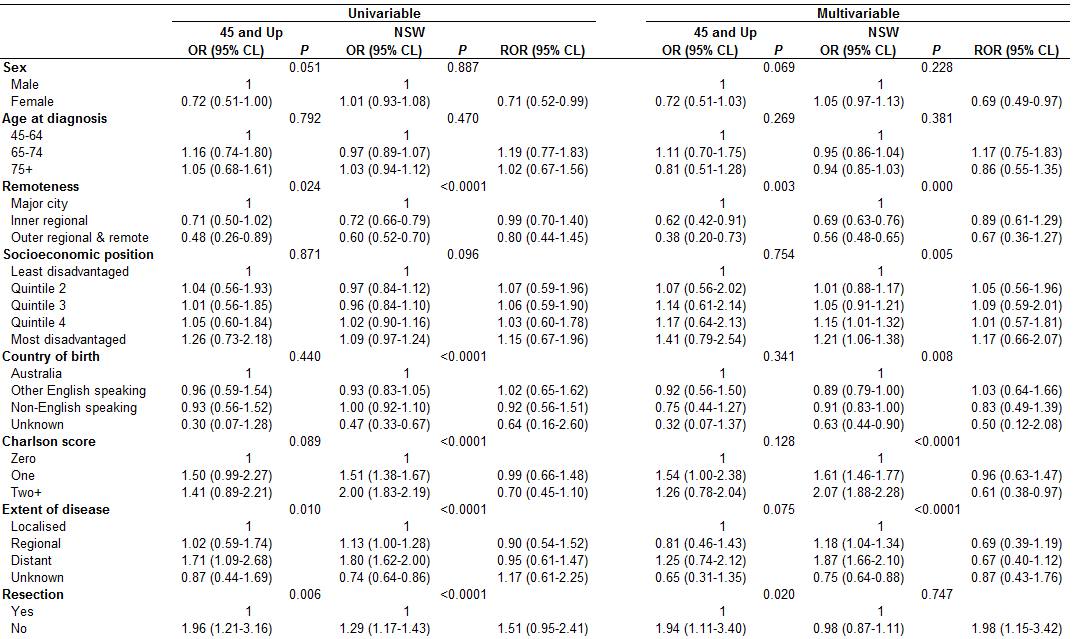


**Table 6: Univariable, multivariable adjusted and relative odds ratios (ROR, 95% confidence limits) of odds of >2 ED attendances in the year after a diagnosis of bowel cancer, 45 and Up Study participants and NSW residents aged ≥45 years**


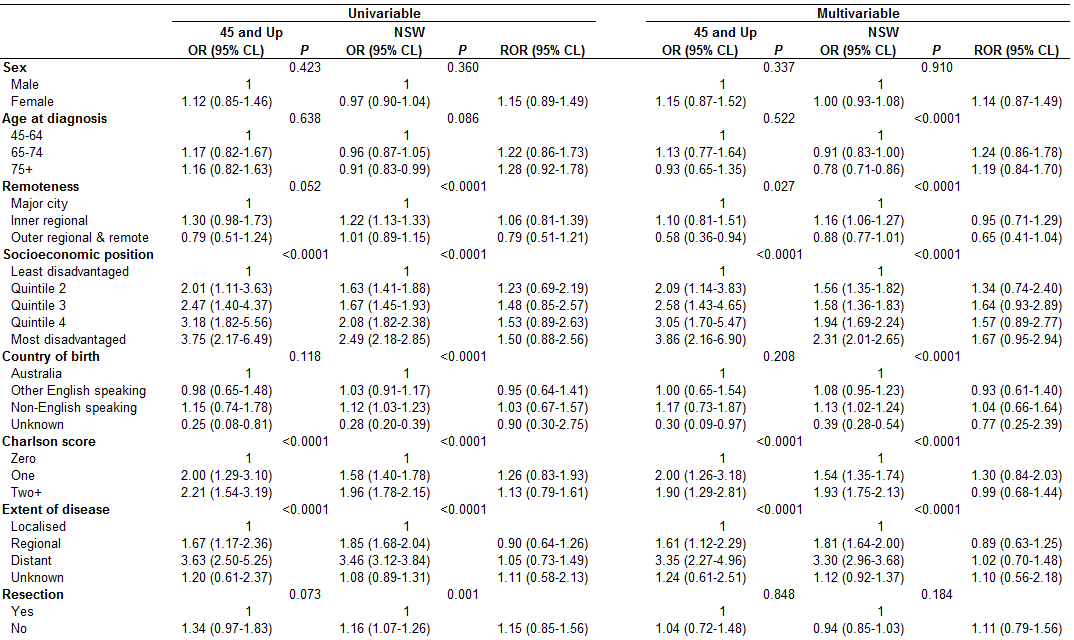


**Table 7: Univariable, multivariable adjusted and relative odds ratios (ROR, 95% confidence limits) of odds of >2 ED attendances in the year after a diagnosis of lung cancer, 45 and Up Study participants and NSW residents aged ≥45 years**


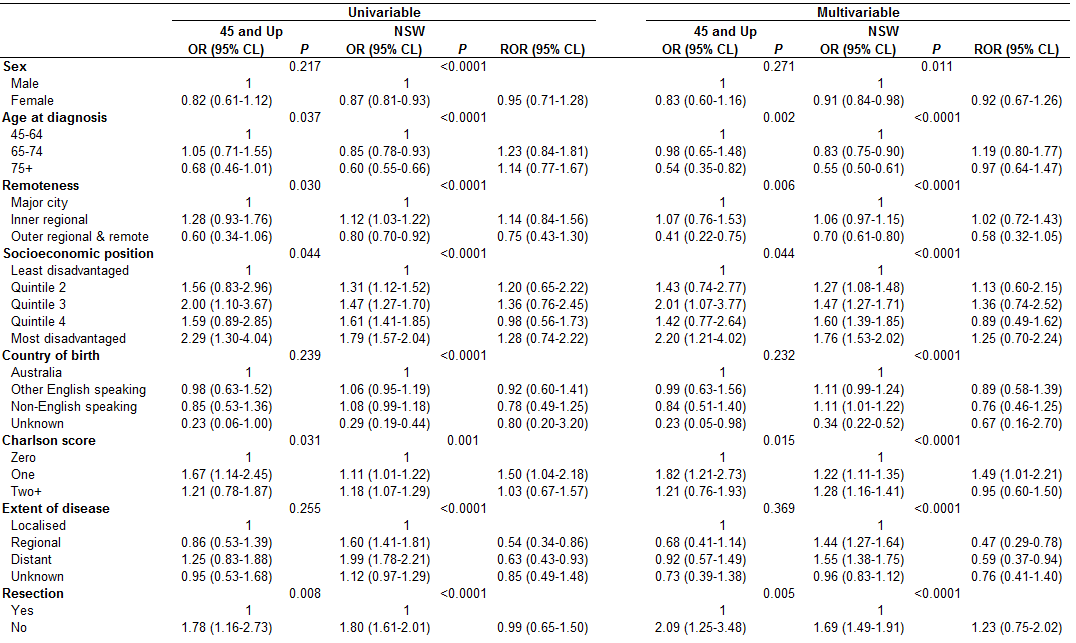


**Table 8: Univariable, multivariable adjusted and relative odds ratios (ROR, 95% confidence limits) of odds of one-year survival following a diagnosis of bowel cancer, 45 and Up Study participants and NSW residents aged ≥45 years**


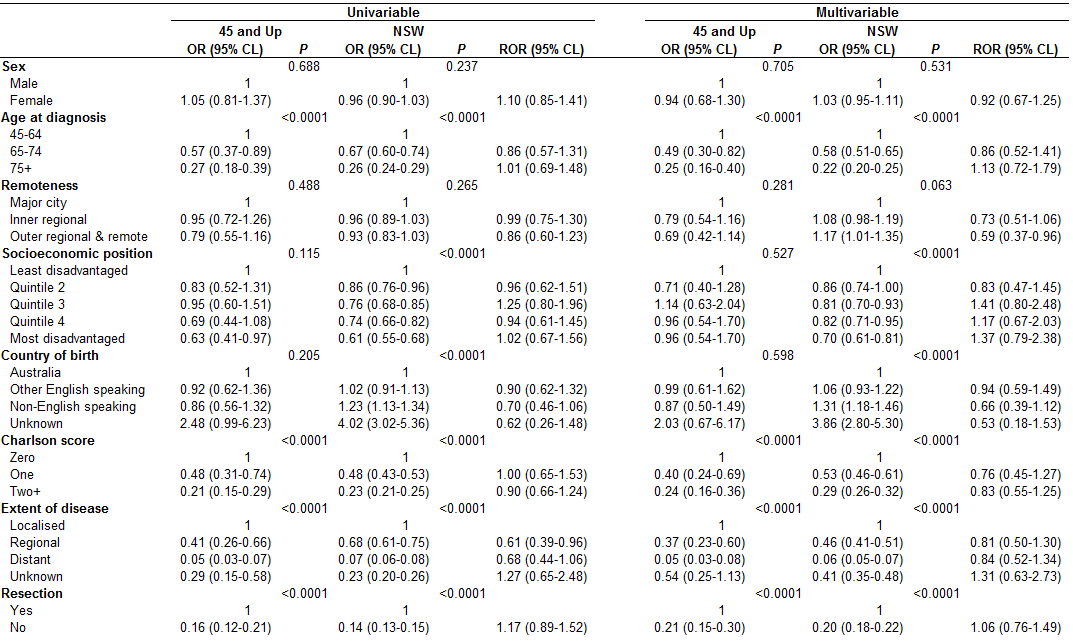


**Table 9: Univariable, multivariable adjusted and relative odds ratios (ROR, 95% confidence limits) of odds of one-year survival following a diagnosis of lung cancer, 45 and Up Study participants and NSW residents aged ≥45 years**


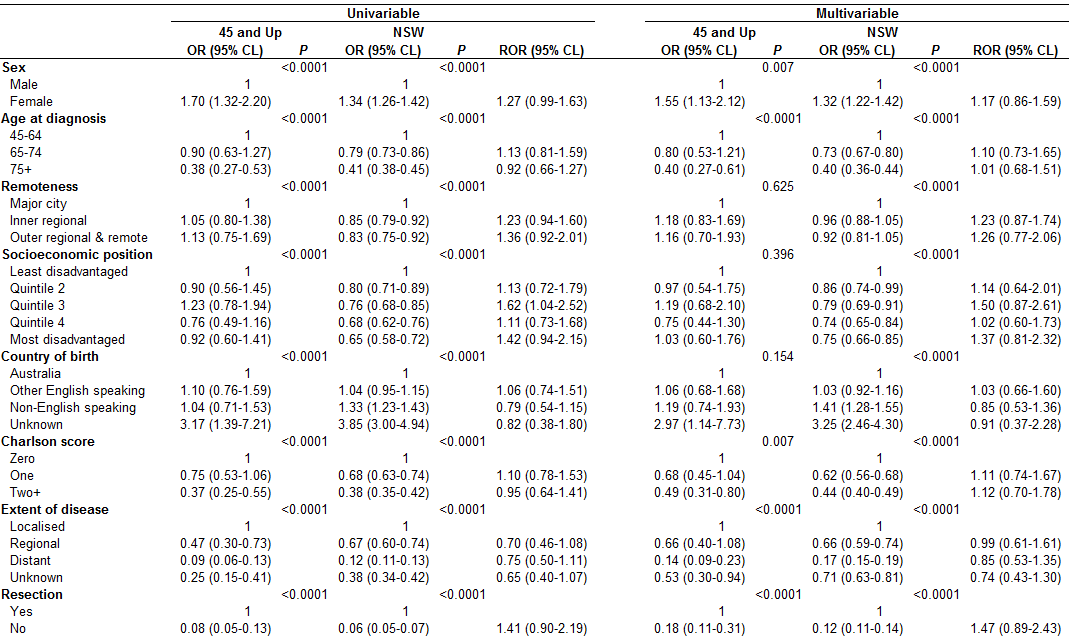

Supplement: Supplementary file 1 — Socioeconomic position by rurality and univariable and multivariable models of health service use outcomes. The additional file contains ratios of relative frequencies for area-based socioeconomic position stratified by rurality (major city; regional and remote) and univariable and multivariable logistic regression models of health service use outcomes (resection; > 4 weeks in hospital; > 2 emergency department attendances; one-year all-cause post-diagnosis survival) for 45 and Up Study participants and NSW residents aged ≥45 years at diagnosis of bowel or lung cancer. (DOCX 610 kb) [file 12874_2018_537_MOESM1_ESM.docx]
